# Some Points in the Treatment of Chorea

**Published:** 1908-01-11

**Authors:** George L. Ranking

**Affiliations:** House Physician at the Royal Berkshire Hospital, Reading.


### The General Practitioner's Column.

[Contributions to this Column are invited, and if accepted will be paid for.]

### SOME POINTS IN THE TREATMENT OF CHOREA.

By GEORGE L. RANKING, B.A.Cantab., M.R.C.S.Eng., L.R.C.P.Lond., House Physician at the Royal Berkshire Hospital, Reading.

The frequency with which chorea is met in childhood, and the distress caused to the patient by undeserved teasing or punishment, make the condition one which, on these grounds alone, it is of the greatest importance to treat, and to treat early.

The severity of chorea varies enormously: from slight hemi-chorea, in which the movements may affect one limb only, to a state in which the patient is never quiet, and continuous violent jerking movements of the whole body are constantly present, possibly with loss of the power of speech and difficulty in swallowing.

The line of treatment must therefore vary slightly in proportion to the severity of the attack, but in every case of chorea the following scheme of treatment will be found to yield satisfactory results:—

### I.—Disciplinary.

There can be little doubt that this forms by far the most important part of the treatment, and in carrying it out the following are some points which should be attended to:

1. The patient should be kept absolutely at rest in bed. Rest in bed will suffice in slight cases to effect a cure without any other treatment. The period over which this must extend varies directly with the severity of the attack; but a minimum of six weeks is in the majority of cases absolutely necessary, whilst two, three, or even four months will be needed in any but the slightest cases. Rest must be absolute, the child being allowed to do nothing for itself. By this means not only are the movements checked, but also the heart, if previously sound, is guarded against damage; whilst if endocarditis be present the heart will, with rest, recover its tone, and the severity of any lesion will be greatly diminished. The importance of rest in bed as a means of cure is shown by the striking difference observed in comparing in- with out-patient cases, the latter often dragging on for months, and finally only being cured by admission as in-patients. The out-patient class entirely fail to appreciate the fact that rest in bed is really the all-important point, and imagine that if the child is kept in bed for a part of the day all that is required has been done.

2. The child should be kept in a room by itself. By this means perfect quiet is ensured. No other children should be allowed in the patient's room. The fewer people the child sees the better. In the event of no special room being available the patient may be screened off.

3. No lessons, books, or toys should be allowed until all movements have ceased.

### II.—Therapeutic Measures.

A. *Treatment by Drugs*.—The fact cannot be sufficiently emphasised that drugs in the treatment of chorea are entirely secondary in importance to rest in bed. The following drugs have been found to give the best results: (1) arsenic, (2) chloretone, (3) zinc compounds, (4) bromides and chloral, (5) salicylates.

1. *Arsenic* may most conveniently be given as the liquor arsenicalis thrice daily, in doses of 3-5 minims, according to the age of the patient. The drug should always be given after food and well diluted. An extra minim may be added every three or four days, until fifteen-minim doses are being given. Signs of overdose, such as diarrhœa, vomiting, or pigmentation of the skin, must be carefully watched for; in the event of these symptoms arising the drug must be stopped, and saline purgatives administered. The majority of children stand arsenic well, and it is certainly the most generally useful drug in this condition.

2. *Chloretone*, or trichlor-tertiary-butyl-alcohol, has lately been given a trial in chorea. In mild cases it certainly seems to quiet the movements quickly, and to be unaccompanied by unpleasant effects; in some cases, however, an irritable papular eruption is met with, which soon disappears when the drug is stopped. In severe cases with violent movements it appears to be of less value; and the following case (which I am permitted to mention by the kindness of Dr. Francis Hawkins) was treated with both chloretone and arsenic. No improvement was shown under treatment with chloretone; whilst as soon as the patient was brought under the influence of arsenic a steady progress was maintained.

B. H., aged 12, schoolgirl, admitted May 7, 1907, with violent and incessant choreic movements of the whole body, had to be constantly watched to prevent her falling out of bed. She was at first treated with chloretone grs. v. thrice daily.

May 10. Movements more violent, quite helpless. Chloretone grs. x. t.d.s.

May 15. No improvement, movements increasing in violence. Has lost the power of speech, unable to swallow.

May 21. Chloretone stopped. Liquor arsenicalis ♏iij. t.d.s. given.

May 28. Movements less violent. Still unable to talk. Slight improvement in swallowing. Arsenic increased to ♏vj. t.d.s.

June 5. Great improvement. Movements considerably less. Power of articulation returning. Power of swallowing much improved. Now taking ♏viij. t.d.s.

June 12-July 3.—During this period the arsenic was increased up to ♏xv. t.d.s.

At the end of this period all choreic movements had ceased, power of speech and swallowing were completely normal, and the child was, except for weakness, due to staying in bed, completely cured.

Whilst it is quite unfair to argue from a single case, the improvement shown under arsenic certainly demonstrated that arsenic will produce good results in a case which has resisted treatment with chloretone.

4. *Bromides and Chloral* will be found to be the most generally useful drugs for overcoming the sleeplessness which is so commonly seen in chorea. The dose must be adjusted to suit the patient's age, and should be given regularly for a few nights, until the condition has begun to quiet down, and regular sleep is obtained.

In the case mentioned above chloral grs. v. and potassium bromide grs. x. were given each night for a fortnight, two hours before the child was settled for the night.

5. *Salicylates.*—These, *per se*, have little or no influence on the movements, and are by no means indicated, unless evidence of very recent rheumatic infection—*e.g.*, subcutaneous nodules—is obtainable. Aceto-salicylic acid has a great reputation amongst many careful clinicians, which is probably to some extent deserved.

*Diet in Chorea.*—The majority of cases, whilst at rest in bed, will do perfectly well on a plain milk diet; and there is no need, at any rate until the movements have become greatly diminished, to go beyond this form of food.

B. *Hydrotherapy* may be carried out by (1) douching, (2) shower baths, (3) wet packs, (4) Chapman's spinal icebag.

The question arises, When ought this part of the treatment to be started? Very violent and excessive movements will often be much relieved either by giving an ordinary wet pack for twenty to thirty minutes every evening, or by constant application of the spinal icebag. Apart from these cases, convalescence will often be hastened by spinal douching, commencing when the movements are first noticed to be lessening in violence. The douche should be given in the evening, the child's back being first sprayed with water at 80-90° F. for a period of five minutes; cold water is then turned on for a similar period, which may be gradually increased until each douche takes twenty minutes. Finally, when the child is said to be cured, it will be wise to warn the friends that relapses are by no means infrequent; and that on the slightest sign of relapse isolation and rest in bed must again be carried out.

